# Treatment of bipolar disorders in older adults: a review

**DOI:** 10.1186/s12991-021-00367-x

**Published:** 2021-09-21

**Authors:** Nemanja Ljubic, Bianca Ueberberg, Heinz Grunze, Hans-Jörg Assion

**Affiliations:** 1 Bereich Forschung & Wissenschaft, LWL-Klinik, Marsbruchstr. 179, 44287 Dortmund, Germany; 2 Psychiatrie Schwäbisch Hall, Ringstraße. 1, 74523 Schwäbisch Hall, Germany; 3 Paracelsus Medical University, Ernst-Nathan Straße 1, 90419 Nuremberg, Germany

**Keywords:** Anticonvulsants, Antipsychotics, Antidepressants, Bipolar depression, Bipolar disorder, Lithium, Mania, Mixed state, Older adults, Old age

## Abstract

**Background:**

Old age bipolar disorder has been an orphan of psychiatric research for a long time despite the fact that bipolar disorder (BD)-I and II together may affect 0.5–1.0% of the elderly. It is also unclear whether aetiology, course of illness and treatment should differ in patients with a first manifestation in older age and patients suffering from a recurrence of a BD known for decades. This narrative review will summarize the current state of knowledge about the epidemiology, clinical features, and treatment of BD in the elderly.

**Methods:**

We conducted a Medline literature search from 1970 to 2021 using MeSH terms “Bipolar Disorder” × “Aged” or “Geriatric” or “Elderly”. Search results were complemented by additional literature retrieved from examining cross references and by hand search in text books.

**Summary of findings:**

Varying cut-off ages have been applied to differentiate old age from adult age BD. Within old age BD, there is a reasonable agreement of distinct entities, early and late-onset BD. They differ to some extent in clinical symptoms, course of illness, and some co-morbidities. Point prevalence of BD in older adults appears slightly lower than in working-age adults, with polarity of episodes shifting towards depression. Psychopharmacological treatment needs to take into account the special aspects of somatic gerontology and the age-related change of pharmacokinetic and pharmacodynamic characteristics. The evidence for commonly used treatments such as lithium, mood-stabilizing antiepileptics, antipsychotics, and antidepressants remains sparse. Preliminary results support a role of ECT as well as psychotherapy and psychosocial interventions in old age BD.

**Conclusions:**

There is an obvious need of further research for all treatment modalities of BD in old age. The focus should be pharmacological and psychosocial approaches, as well as their combination, and the role of physical treatment modalities such as ECT.

## Background

Recent studies assume that 25% of patients with BD in the western world are over 60 years old [1].

However, diagnosing affective syndromes in older people can be difficult. The incidence of affective disorders in old age can be easily underestimated, as the established diagnostic criteria of the International Classification of Diseases (ICD-10) [2] and the Diagnostic and Statistical Manual of Mental Disorders (DSM-5) [3] have limited applicability to this population. Frail patients in nursing homes, for example, may be misjudged, as physical illnesses may produce symptoms not only of depression (e.g., rapid exhaustion), but also of mania [4].

The increased awareness of the public of BD has led to a growth in the number of specialized services for this illness but seems to have missed the elderly out. In general, the treatment of older bipolar patients can be more complex than in younger patients and faces different needs given the higher prevalence of medical comorbidities, sensitivity to treatment-related adverse effects, and complex psychosocial challenges [1]. In this regard, the Task Force report of the *International Society of Bipolar Disorders* (ISBD) on older-age BD [1] stresses the importance of developing specific psychosocial treatment programs for older people with severe mental illnesses, such as the *Helping Older People Experience Success* (HOPES) program [5], which proved to be superior to standard care.

Surveys show that hardly any national or international guidelines on BD deal with the topic of "advanced age" [6, 7], in part attributable to the general lack of evidence from clinical trials in the elderly [8]. Bipolar guidelines usually mention old age BD only in the context of reduced tolerability and safety and stress the need of more research specific to the diagnosis and specialized services for this population [9, 10]. The aim of this narrative review is to summarize the current state of knowledge about the epidemiology, clinical features, and treatment of BD in the elderly.

## Methods

We conducted a Medline search on April 20, 2021 using MeSH terms “Bipolar Disorder” × “Aged” or “Geriatric” or “Elderly”. Results were further categorized by adding additional search terms: “etiology” or “aetiology”, “treatment”, “randomized”, “mania” or “manic”, “depression” or “depressive”, “mixed”. Additional literature was retrieved examining cross references and by hand search in text books.

## Summary of findings

### Definition of old age bipolar disorder

There is no clear consensus of what constitutes "advanced age" in BD. From the perspective of geriatric psychiatry services, age limits of 60 or 65 years are discussed; post hoc analyses of phase three medications trials used 55 years as cut-off [11], and from the lifetime perspective of bipolar disorder, an age limit of 50 years—sometimes even 40 years—has been proposed because of the early age of onset of the disorder [1]. Hence, it was recognized that older adults with BD may comprise four distinct groups of individuals, which are detailed in Table 1.

**Table 1:** Defining old age bipolar disorder [113]

Early-onset bipolar disorders	"Old" bipolar patients with early onset of the disease
"Real" late-onset bipolar disorders	In some patients, bipolar disorder actually manifests itself for the first time after the age of 50
Late-onset bipolar disorder with earlier pseudo-unipolar course	A manic, hypomanic or mixed episode develops for the first time after the age of 50, although depressive episodes had already occurred before the age of 50
Secondary manias	As a result of a somatic disease or medication- or drug side effects lead to the development of a manic syndrome

*Early-onset bipolar disorder* In most cases the age of onset of BD is in adolescence and early adulthood. Even though mortality in younger people with BD due to natural and unnatural causes is higher than in the general population [7], the vast majority of bipolar patients reaches an age beyond 60. Since BD constitutes a chromic and lifelong disorder, hypomanic, manic, depressive and mixed episodes are observed in all age groups.

*"Real" late-onset bipolar disorders* A small proportion of BD manifests itself late in life. In such cases—by definition—a first hypomanic, manic or mixed episode occurs after the age of 50. Obviously, this makes it difficult to distinguish it from a late-onset BD with a previous pseudo-unipolar course; often earlier depressive episodes are not remembered and reported. Mild earlier hypomanic or mildly mixed episodes may also be overlooked. Temperament might also be another confounder of diagnosis if there are temperamental peculiarities, such as hyperthymic, cyclothymic, irritable or depressive temperament [12].

*Late-onset bipolar disorders with a previous "pseudo-unipolar" course* Some patients develop hypomanic, manic or mixed episodes after the age of 50, without any previous episodes having been observed. However, they had suffered from at least one depressive episode before the age of 50. This means that now the entire course of the disease needs to considered as bipolar. The supposedly unipolar course before the age of 50 can be described in retrospect as "pseudo-unipolar" [13]. Jules Angst has shown in the prospective Zurich cohort study that manic episodes manifest themselves with a more or less constant risk throughout the life of a person with recurrent depression—even after the age of 70—with a conversion rate of about 1–1.5% per year [14]. Consequently, a manic, hypomanic or mixed episode can also occur for the first time "in old age". In line with this, a large population-based study in the UK reported that approximately 10% of all bipolar patients experience their first manic episode after aged 45 years [15].

*Secondary manias* Secondary manias are manic syndromes resulting from somatic diseases or pharmacological (side) effects [16]. There are numerous causes of such secondary manias. Besides drugs (e.g., cortisone, antibiotics, especially gyrase inhibitors, chemotherapeutics), neurovascular and systemic causes are also known [17, 18]. In addition, secondary mania may occur in a person with no history of mood disorder and may be associated with systemic infections, such as influenza, HIV, St Louis type A encephalitis and Q fever, and other medical or neurological aetiologies [19]. As older people are more likely to be multimorbid, they obviously have more risk factors for developing secondary mania.

### Epidemiology

Although still common in older people, the point prevalence of BD decreases with age [20]. BD-I and II together may affect 0.5–1.0% of the elderly [21, 22] with approximately 0.4% suffering from BD-I [23–26]. Within the elderly population seeking medical care, BD patients account for 6% of outpatients, 8–10% of geriatric hospital admissions, and 17% of elderly in psychiatric emergency rooms, with more than two-thirds being females [24, 27, 28]. In different treatment settings, point prevalence of manic episodes was reported to range from 1.6% to 50%—with a mean of 6% [29]. However, these results come from unrepresentative samples and are therefore difficult to generalize. As with most geriatric psychiatric samples and unlike in BD-I at a younger age, females predominate (2:1 to 3:1).

There is no clear evidence that BD at the age of 65 years and older follow a significantly different psychopathological course than BD at younger ages, apart from the consequences of the physiological aging processes [30]. Polarity of BD appears to shift with older age towards depression, and consecutively less time spent in manic or mixed states [31]. The so-called *Kindling hypothesis* that the intervals between episodes become shorter and shorter in the course of BD is still controversial. Several studies have described a decrease of inter-episode intervals with successive episodes [32–34]. However, two analyses of the Zurich study produced contradictory results depending on the methodology used [35, 36]. Accordingly, a recent task force report of the International Society for Bipolar Disorders (ISBD) on BD of old age concludes that it remains uncertain whether or not a progression of symptoms occurs with age [1]. The fact that suicide rates decrease in the elderly with BD compared to people with BD under 35 might be well explained by a "survival effect" [1].

According to the Zurich study, BD accounts for 19% of affective disorders in the elderly [14]. In direct comparison to recurrent unipolar depression, BD of older age showed a worse course and outcome [30, 37, 38]. There is evidence that older patients with BD more often suffer from cognitive decline [38]. A recent study reported that longer duration of illness is related to lower gray matter volume [39]. Neurocognitive impairment is seen in approximately one-third of elderly BD patients [40] and it is believed that it may reflect some kind of encephalopathy [41]. However, it remains unclear whether this assumed encephalopathy is static or a progressive process. A controlled prospective 5-year study found that older bipolar patients had more cognitive impairment at baseline than a healthy control group. Over the 5-year course of the study, however, the cognitive decline was not different between both groups [42].

Comorbidity with other psychiatric disorders seems to decline with age when comparing working-age and old age BD patients [43–45], but there is some evidence that neurological and other somatic disorders are more common among older bipolar patients compared to the elderly general population [45, 46]. Comparing elderly patients with BD and with unipolar depression, Shulman and colleagues found that 36% of bipolar patients but only 8% of patients with recurrent depressive disorders had a neurological disorder in old age [47]. This could be due to different aetiologies (vascular, traumatic or degenerative), so it is debatable whether a relevant part of this sample probably suffered from "secondary manias" caused by a somatic disorder with neurological and psychological symptoms. In fact, more frequent extrapyramidal motor symptoms and more cognitive deficits, paralleled by a decline of social functioning [48], have been reported for patients with late-onset BD than for elderly patients with early-onset bipolar disorders [49–51]. The prognosis of patients with neurological or other somatic illnesses developing secondary mania differs from the prognosis of "primary" forms of BD in old age [52]. In general, the occurrence of manic symptoms in older people seems to predict an inferior course compared to depressive symptoms only [53].

### Clinical features and diagnosis

BD across age groups is characterized as a chronic condition with recurrent mood episodes. Recent studies have found that clinical features and, as a consequence, treatment approaches of older bipolar patients differ from those of younger patients [54] (see Table 2). Generally, the evaluation of manic symptoms or episodes in an older adult requires a thorough differential diagnosis to accurately determine the cause and to guide appropriate treatment. The observed manic syndrome of an older person might be a "secondary mania", e.g., the consequence of a physical illness, a medication- or drug effect, or a "primary" manifestation of BD [16]. Ultimately, this question can only be answered if the longitudinal course of the disorder is taken into account and also requires an evaluation of interfering or confirmative factors such as the current medication, the somatic findings, the psychopathology, and also the family history.

**Table 2:** Characteristics of bipolar disorder in older versus younger populations. Modified from [114]

Characteristics	BD in older adults		BD in younger adults
Comorbidity with physical illnesses	Higher		Lower
Psychosocial events as a trigger	More		Less
Cognitive dysfunction	Higher		Lower
Symptoms of mania and depression	Similar		Similar
Prevalence of depressive episodes	Higher		Lower
Severity of depressive episodes	Lower		Higher
Antidepressant use	Higher		Lower
Secondary mania	Higher		Lower
Family history of mood disorder	Lower		Higher
Comorbidity with mental illnesses (all)	Lower		Higher
Substance use disorders	Lower		Higher
Personality disorders	Lower		Higher
Attention deficit hyperactivity disorders	Lower		Higher

The symptomatology of depression in the elderly appears similar between unipolar and bipolar patients, and most data stems from research in mixed populations. Depressive episodes in older adults may be associated with more sleep disturbance, fatigue, psychomotor retardation, and hopelessness about the future than in younger adults with the same disorder [55]. Common complaints of elderly patients with depression are also poor memory and concentration [56], and slower cognitive processing speed and executive dysfunction confounding with dementia, called pseudodementia, have been reported [57]. Depression in old age is frequently associated with neurological comorbidities as well, like Parkinson's disease and stroke.

Considering the mentioned facts, the diagnosis of mania in old age can present a challenge for psychiatrists, particularly in distinguishing mania from dementia, delirium, or agitated depression. The following psychiatric symptoms have been reported in Alzheimer’s disease in common with the profile observed in late-onset BD: agitation, euphoria, disinhibition overactivity without agitation, aggression, affective liability, dysphoria, apathy, impaired self-regulation, and psychosis [58]. Therefore, the following guidance may be helpful for the differential diagnosis in patients presenting with a combination of manic and cognitive symptoms:

- The onset of a manic episode may be signalled by a rapid decline in cognitive functioning in a patient who has dementia, along with fluctuations in mood, energy, and sleep;
- Depressive symptoms are common in older patients who are manic (mixed states);
- Different from BD, dementia is typically associated with focal neurological findings, such as aphasia, apraxia, or impaired visuospatial functioning;
- It is more typical for dementia or delirium to be associated with night-time agitation and confusion;
- A positive family history of BD is supportive for diagnosis, but a negative family history of BD may be unreliable, as family members may have received a diagnosis (or misdiagnosis) before the modern diagnostic classification.

However, the literature is still inconclusive concerning the presence or absence of clinical patterns clearly distinguishing old age BD from younger patients, as well as early vs. late-onset BD within the group of elderly BD patients [24, 59–66].

### Pharmacological treatment

The initial therapeutic approach must consider the multiple aspects that differentiate the clinical features of BD in old age from findings in younger population, and take into account the special aspects of somatic gerontology [20]. For the psychopharmacological therapy, the age-related change of pharmacokinetic and pharmacodynamic characteristics (such as distribution volume, protein binding, metabolism) must be taken into account. The altered cellular functions (reduced enzyme activities) frequently require adaptation using lower doses in the elderly. Especially the microsomal metabolism involving CYP3A4, the main metabolic pathway of frequently used antidepressants such as sertraline and citalopram, diminishes with age. Of note, however, there appears to be minimal age-related changes in CYP2D6 function which is relevant for metabolizing several other antidepressants, such as several tricyclics, fluoxetine and the SNRIs duloxetine and venlafaxine. The same is true for phase II metabolism (e.g., glucuronidation, sulfation, or acetylation) which appears unchanged in the elderly [67].

With age-related decreases in total albumin, plasma binding can be diminished and significantly decreases with diseases and malnutrition [68]. This affects the ratio of bound vs. unbound (active) drug in commonly used medication such as lorazepam and valproate, resulting in changes of speed of release and, by this, possibly efficacy and adverse reactions.

**Fig. 1:**
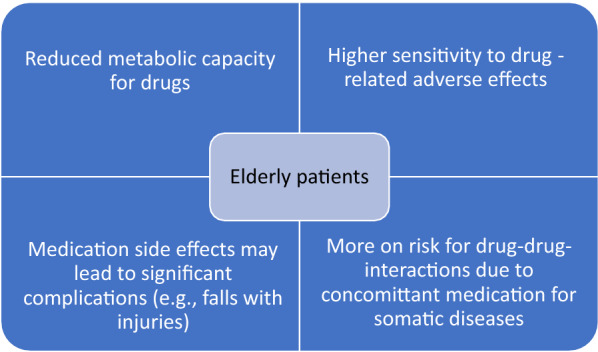
Key clinical issues among old age patients prescribed psychotropic medication (modified from [82])

Adverse drug reactions increase with age, even at lower drug concentrations, including tardive dyskinesia, dizziness, and sedation. Also with age, the homeostatic reserve is fading (e.g., impaired thermoregulation or thirst regulation), again resulting in decreased tolerance to adverse effects. In addition, an elevated probability of drug interactions exists given the larger number of medications taken, and an increased number of medications may also cause difficulties with adherence, especially in those with incipient cognitive impairment [67]. Figure 1 presents some of the key clinical issues among elderly patients prescribed psychotropic medication.

The paucity of rigorous research and published studies on the psychopharmacological treatment of BD in old age presents a huge problem in the management of this disorder. The main reason for the limited research on pharmacological treatments is related to the fact that old age BD patients are at a higher risk of medical complications [69] and therefore willingly excluded from confirmative phase 3 studies. As a matter of fact, a recent systematic literature search revealed that not a single double-blind placebo-controlled study has been conducted exclusively in an old age BD population. Looking into comparator trials, Fountoulakis (personal communication) identified altogether three studies in acute mania (comparing lithium to valproate [70], lithium to memantine [71], and a post hoc analysis of pooled data of patients aged ≥ 55 years from two quetiapine monotherapy clinical trials [72], and one study each in bipolar depression (a post hoc analysis of two placebo-controlled, 6-week, randomized, double-blind studies with lurasidone [73]) and maintenance (a post hoc analysis of two double-blind maintenance studies comparing lamotrigine, lithium and placebo [11]). Despite the paucity of evidence, some researchers tried to sum up the available evidence on the management and treatment of BD in old age, e.g., [11, 52, 54, 74, 75]. Summarizing these efforts, and in the absence of contradicting evidence, current guidance concludes that first-line treatment of old age BD should be similar to that for adult BD, with specific attention to vulnerability to side effects and somatic comorbidities [20].

#### Lithium

Lithium is considered an effective therapy for manic and depressive episodes and relapse prevention of bipolar disorders [69, 76]. This appraisal is, by large, based on extrapolation from adult studies, naturalistic studies (e.g., [77]), a single controlled trial in acute mania (vs. valproic acid) and a post hoc analysis of maintenance trials. In the acute mania trial, lithium was superior to valproic acid in YMRS score change from baseline (*p* < 0.001 at endpoint (week 9)). Compared to valproic acid, there was also a superior response to lithium in patients with YMRS > 30 [70]. Post hoc analysis of the maintenance studies showed that lithium significantly delayed time to intervention for mania/hypomania/mixed episode in comparison to placebo, but not for time to intervention for depression [11]. Obeying the pharmacodynamic and pharmacokinetic factors that change with age and the higher risk of side effects and interactions, a treatment trial with lithium can be recommended, even more so for primary than secondary manias [78]. With comparable resorption of lithium, the distribution volume is higher in old age, but the renal clearance is reduced. The twofold prolonged elimination requires a lower dose of one-third to one-half. With close-meshed control of the serum level, a slow increase in dosage is recommended. Neurotoxic effects (e.g., loss of consciousness, tremor, ataxia) are usually dose-dependent, but can also be independent of the dose level [79]. Interactions with other somatic medication also play a more prominent role in old age BD; for example, diuretics of the thiazide type, angiotensin conversion enzyme (ACE) inhibitors and non-steroidal anti-inflammatory drugs (NSAID) increase, whereas carbonic anhydrase inhibitors decrease lithium plasma levels [80]. For safety reasons, lower serum levels between 0.4 and 0.6 mmol/l are usually recommended for prophylactic treatment in old age; however, the evidence for this range and even lower levels is weak. As with working-age BD patients, manic episodes usually require higher doses.

#### Valproic acid and derivatives

The efficacy of valproic acid (synonymous valproate, valproate, dipropyl acetate) or its derivatives (valpromide, divalproex) has been only tested in the already cited controlled trial against lithium [70], with valproic acid showing inferior response compared to lithium. Based on retrospective chart analysis [81], case reports [82] and in the absence of other medication with high quality evidence, it is, however, frequently used in old age BD. The fairly good tolerability is one of the reasons for the frequent use of the substance. Valproic acid is not metabolized by the cytochrome C system, but by glucuronidation and β-oxidation. For dosing, both the higher proportion of free valproic acid (without binding to proteins) in old age and the longer elimination half-life should be taken into account [83]. Fatigue and occasional gastrointestinal complaints, tremors and gait disturbances may occur, as well as a reduction in platelets. Serious and very rare are encephalopathy or damage to the liver [84]. For the treatment of mania in adult age, serum concentrations above 90 ug/ml should be targeted [85] but no data have been generated so far for geriatric mania. There are no controlled maintenance data for valproic acid in old age BD, and data from adult age trials are inconclusive [86]. The usually recommended serum concentration above 45 mg/l for maintenance is not based on evidence in BD patients, but in epilepsy patients. For adult BD patients, a post hoc analysis of the failed valproic acid vs. lithium vs placebo study [87] showed that valproic acid plasma levels between 75 and 99.9 mg/l valproic acid were significantly better than placebo in secondary outcomes (discontinuation for any reason, mania and depression) [88]. Again, no data specifically for old age BD have been generated so far.

#### Carbamazepine

Carbamazepine is only a subordinated choice in the treatment of old age BD because of its autoinduction of metabolism, frequent interactions and possible neurotoxic effects. The effect on cytochrome (CYP) P450 (induction of 2D6) leads to frequent interactions with concomitant medication as approximately 25% of all medication are metabolized via CYP 2D6, including several antipsychotics, antidepressant and anticonvulsants. Undesirable neurotoxic effects (blurred vision, diplopic images, nystagmus, confusion, agitation) are frequent in higher dosages, and changes in blood count (agranulocytosis, aplastic anaemia), allergies, hyponatremia or urinary retention may occur more frequently than with other mood stabilizers [89], and especially blood dyscrasias with carbamazepine may occur more frequently in the elderly [90]. A serum level between 4 and 10 mg/l has been established as a suitable therapeutic level in epilepsies, but, again, robust evidence for serum levels in BD has not been published so far.

#### Lamotrigine

With its good tolerability and safety profile, lamotrigine appears as an attractive choice for treatment in the elderly with BD [91]. The predominant route of lamotrigine elimination is hepatic metabolism. Metabolic inactivation is catalysed by the UDP‐glucuronosyltransferases (UGT), and the main metabolite lamotrigine‐n‐2‐glucuronide is excreted by the kidneys. Oral clearance of lamotrigine appears reduced in the elderly by 20–35% and dose-adaption may be needed [92].

An open study of lamotrigine add-on to lithium or valproic acid suggested antidepressant efficacy in geriatric BD patients [93]. In a pooled analysis of two controlled studies, lamotrigine reduced time to relapse into a depressive episode [94]. Post hoc analysis of these studies confirmed that this effect is also true in patients ≥ 55 years [11]. The possibility of allergic reactions requires a low starting dose with a slow increase in total daily dose. A reduction of the daily dose is necessary in cases of liver or kidney dysfunction. A serum level between 1.5 and 10 mg/l is considered a suitable therapeutic level in epilepsies [95], whereas a robust therapeutic range has never been established in BD. A small maintenance study suggests that in rapid cycling patients serum levels ≥ 5 mg/l should be targeted [96].

#### Antipsychotics

Antipsychotics are a first-line treatment in acute mania; a combination with benzodiazepines is common practice. However, their usage in geriatric patients is controversial as they may increase mortality due to cardio- and cerebrovascular events, at least in patients with dementia (FDA Black box warning from 2005 [97]). Factors increasing the risk of stroke (including high blood pressure, TIA, diabetes mellitus, tobacco consumption and elevated cholesterol) need regular check-ups. First-generation antipsychotics (FGA, e.g., haloperidol) have been frequently used, but have hardly been studied for their effectiveness in acute mania or preventing relapse at an older age. The increased risk of extrapyramidal side effects (EPSE) (tremor, parkinsonism) in long-term use of antipsychotics, especially in old age, is well known [98, 99]. With rapid dose escalation as needed in acute mania, the risk of oversedation and falls is higher than in working-age patients. Regarding EPSE, second-generation antipsychotics (SGA) have proven to be more beneficial [100], and in contrast to FGA, several SGA also have demonstrated antidepressant and mood-stabilizing long-term effects in adult BD [86, 101]. For acute mania, post hoc evidence from a controlled study exists for quetiapine, and open label trials and case series suggest also efficacy for asenapine, aripiprazole, clozapine and risperidone [54]. Studies of SGA in bipolar depression and maintenance in old age BD, however, are lacking. Metabolic side effects (hyperlipidaemia, diabetes mellitus) and cardiac side effects are well known with SGA and need close monitoring.

In general, antipsychotics have a beneficial effect on BD-related motor restlessness, anxiety, irritability or agitation. Strong sedative effects and impairment of memory performance can be disadvantageous. EPSE including frequent akathisia, as experienced with some antipsychotics, are associated with a higher risk of falls and fractures.

Substances with an anticholinergic component (particularly low-potency antipsychotics, such as levomepromazine) are not recommended for older patients because of their negative impact on neuropsychological performance. In addition, urinary retention is a possible adverse anticholinergic effect in older males.

#### Antidepressants

Antidepressants can provide short-term benefit, but may increase the risk of mania and rapid cycling in the long-term in BD-I patients with relevant risk factors [102]. On the other hand, acute responsiveness to antidepressant might also predict long-term benefit without an increased risk of mania [86], and the natural odds of a manic recurrence also decreases with age [31]. In addition, the newer generation of antidepressants is usually well tolerated and safe, and might have additional benefits for health, e.g., reduction of cardiovascular mortality with SSRIs [103]. Hence, whether to continue or discontinue antidepressants after an acute episode is a very individual decision-making process. In those patients on risk of a treatment emergent affective switch or rapid cycling, mood stabilizers (lamotrigine, lithium, valproic acid) and several SGA should be considered first before antidepressants are used.

### Non-pharmacological treatments

Several studies support combined psychosocial and pharmacological treatments in older adults with depression (e.g., [5, 104]). The study by Frank and colleagues also demonstrated that elderly patients are as responsive to psychotherapy as working-age adults [104]. According to Bartels and Pratt, older adults with serious psychiatric illness receiving psychosocial interventions showed clear improvement in their quality of life [105]. A similar finding was also reported by Depp et al. who found that their psychosocial program is effective in improving adherence, depressive symptoms, and quality of life as well [106]. In addition, this integrative model may train preventive strategies, including management of stress, attention to biological rhythms, and improved medication compliance, as well as reparative measures to deal with the interpersonal, social, and practical aftermath of a manic or depressive episode.

As a physical treatment modality in old age BD, electroconvulsive therapy (ECT) constitutes an alternative treatment modality to treat both mania and depression [107] and can also be used in continuation treatment [108]. Robust evidence for efficacy of ECT in old age BD is largely absent, but studies in older patients with unipolar depression have demonstrated that not only mood, but also cognitive symptoms may improve with ECT, mediated by improvements in focus and depressive mood symptoms [109–111]. Furthermore, Greenberg and Kellner proposed that, similar to working-age patients, the use of ECT in elderly with BD may be most useful for patients with treatment refractoriness to medication, and in those refusing fluids and foods, or individuals with severe suicidal thoughts [112]. However, additional research regarding the effectiveness and safety of ECT in the elderly with BD is demanded.

Table 3 summarizes the evidence for the different treatment modalities in old age BD.

**Table 3:** Summary of the evidence for frequently used medication, physical treatments and psycho/psychosocial therapies in old age BD

Treatment	Mania	Bipolar depression	Maintenance	Notes
Lithium	+	0	+	For mania: EP-ABD, [70]; For maintenance: LGE
Valproate	+	0	0	For mania: LGE
Lamotrigine	–	+	+	For depression and maintenance: LGE
Antipsychotics	+	0	0	For mania: LGE for quetiapine, asenapine, risperidone, aripiprazole, clozapine For depression and maintenance: EP-ABD
Antidepressants	–	0	0	For depression and maintenance: EP-ABD
ECT	+	+	0	All EP-ABD or trials in geriatric unipolar depressed patients
Psychotherapy and psychosocial treatment	–	+	+	EP-ABD or LGE

The grading is based on the evidence reported in this review and only reflects the opinion and judgement of the authors

Grade of recommendation: +: recommended, 0: conflicting evidence or unfavourable risk/benefits ratio; may be considered in selected patients, –: not recommended

EP-ABD: Extrapolated from controlled studies in 18–65 year-old adult BD patients

LGE: Low grade evidence such as post hoc analysis, open studies, retrospective chart reviews

[..] Reference to controlled study in old age BD

## Conclusion

In conclusion, there is a tremendous need of further research into the treatment of BD in older adults. This up-to-date review points out that focus should be on both, pharmaceutical and psychosocial approaches, as well as their combination. For the time being, the take-home message for clinicians is that they can rely on the evidence for pharmacological and physical treatments derived from controlled studies in working-age adults while being aware of the changes in metabolism and sensitivity to side effects in older adults. As far as we can say right now, differences in efficacy of medication between working–age adults with bipolar disorder and older adults are, if at all, subtle; meaning that individual parameters such as physical health issues, interaction potential with somatic medication and ease of use play a more prominent role when choosing the appropriate medication. With the little data available, it appears that most established psychotherapies for bipolar disorder are also effective in older adults. When initiating a specific psychotherapy, not only the cognitive capabilities need to be taken into account, but also the individual life—and social circumstances that arise with aging, e.g., retirement, family support and role within family, and other sources of psychosocial support.

## Data Availability

All data given in this review have been previously published and are in the public domain.

## Abbreviations

ACE: Angiotensin conversion enzyme
BD: Bipolar disorder
ECT: Electroconvulsive therapy
ICD-10: International Classification of Diseases
DSM-5: Diagnostic and Statistical Manual of Mental Disorders fifth revision
EPSE: Extrapyramidal side effects
FDA: U.S. Food and Drug Administration
FGA: First-generation antipsychotics
HOPES: Helping Older People Experience Success
ISBD: International Society for Bipolar Disorders
NSAID: Non-steroidal anti-inflammatory drugs
SGA: Second-generation antipsychotics
